# Natural Pigments and Cyclodextrins: Overview on a Synergistic Approach to Encapsulation

**DOI:** 10.1111/1541-4337.70620

**Published:** 2026-08-27

**Authors:** Chiara Pippolini, Ilaria Benucci, Claudio Lombardelli, Marco Esti

**Affiliations:** ^1^ Department of Agriculture and Forestry Science (DAFNE) Tuscia University Viterbo Italy

**Keywords:** cyclodextrins, encapsulation, inclusion complex, natural dyes, stability

## Abstract

The growing demand for natural dyes in the food industry has stimulated research into strategies aimed at improving their stability, solubility, and bioavailability. This review examines the synergistic use of cyclodextrins (CDs) as encapsulating agents for natural pigments, highlighting their ability to form inclusion complexes that protect pigments from various external factors. Following an overview of the structure, chemistry, and functionalization of CDs, their role in stabilizing different classes of natural pigments is discussed. Among the various types, β‐CD emerges as the most widely used due to its optimal cavity size and commercial availability. The main encapsulation techniques, along with the analytical methods used to characterize the resulting complexes, are also presented. Finally, several studies are reviewed, confirming that CDs significantly enhance the physicochemical properties and solubility of natural pigments, thereby supporting their application in food matrices with results comparable to those of synthetic dyes.

## 1 Introduction

Natural pigments are increasingly attracting interest as alternatives to synthetic colorants due to growing consumer demand for clean‐label and naturally derived food ingredients. In addition to their coloring properties, many natural pigments exhibit bioactive functions and potential health benefits (Lombardelli et al. 2020). However, their application in food systems remains limited because they are often highly sensitive to environmental factors such as light, oxygen, temperature, and pH variations, which may lead to color loss and degradation during processing and storage (Benucci, Lombardelli, et al. 2022). Consequently, considerable research efforts have been devoted to the development of stabilization strategies capable of improving the physicochemical properties, stability, and bioavailability of these compounds (Mazzocchi et al. 2025). Among the various approaches proposed, encapsulation has emerged as one of the most promising technologies, with cyclodextrins (CDs) attracting particular attention due to their ability to form inclusion complexes that protect sensitive molecules while enhancing their solubility and functionality.

CDs are produced as crystalline, homogeneous, nonhygroscopic cyclic oligosaccharides, through starch biodegradation using α‐glucanotransferase enzymes. The most common ones, also referred to as first‐generation or progenitor CDs, consist of six, seven, or eight glucopyranose subunits and are known as α‐CD, β‐CD, and γ‐CD, respectively (Table 1). These molecules have 18, 21, and 24 hydroxyl groups (−OH; Kali et al. 2024).

**TABLE 1:** Key structural parameters of the three natural cyclodextrins (CDs; α, β, γ; Fuenmayor et al. 2021).

Type of CD	Number of glucopyranose units	Outer diameter (nm)	Inner diameter (nm)	Height (nm)	Internal volume (nm^3^)
α‐CD	6	1.37	0.57	0.78	0.174
β‐CD	7	1.53	0.78	0.78	0.262
γ‐CD	8	1.69	0.95	0.78	0.427

Besides the three main types, larger forms of cyclic oligosaccharides also exist, containing nine to 12 glucose units and referred to as δ‐, ε‐, κ‐, and μ‐CDs (Kali et al. 2024).

Production volumes of CDs exceed 10,000 metric tons annually, with β‐CD representing approximately 70%, followed by α‐CD (15%), γ‐CD (5%), and other derivatives (10%; Hu et al. 2022).

### 1.1 Functionalization and Derivatization of CDs

In addition to their natural forms, numerous chemical derivatives of CDs have been developed to improve their physicochemical properties and broaden their potential applications. Modifications can occur through two different methods: enzymatic and chemical. Enzymatic methods allow the attachment of monosaccharides or oligosaccharides to CDs, exploiting the action of enzymes such as CD glucanotransferase (CGTase) and pullulanase. The result is the formation of branched CDs, such as maltosylated and glycosylated CDs (Z. Zhang et al. 2024).

Chemical modifications typically involve the introduction of functional groups through substitution reactions. Due to their nucleophilic nature, the hydroxyl groups on the CD surface can react with electrophilic compounds such as isocyanates, alkyl halides, acyl derivatives, or epoxides, leading to the formation of ether or ester bonds and thus altering the CD structure (Dhiman and Bhatia 2020).

CDs can also be functionalized through reactions with nucleophilic molecules like thiols, amines, or halides, following appropriate chemical activation. These structural modifications, primarily occurring at the hydroxyl groups, can significantly increase water solubility, in some cases up to 25–30 times that of native CDs, and enhance their versatility in fields such as pharmaceuticals, cosmetics, agri‐food, and materials industry (Topuz and Uyar 2024).

Some of the most commonly used derivatives include hydroxypropyl‐β‐CD (HP‐β‐CD), methyl‐β‐CD (M‐β‐CD), hydroxypropyl‐γ‐CD (HP‐γ‐CD), and carboxymethyl‐β‐CD (CM‐β‐CD), which are widely employed in fields such as the pharmaceutical sector, as well as in cosmetics and the food industry (Y. Zhang et al. 2021; Ferreira et al. 2023; Puskás et al. 2023). The most commonly used CD derivatives and their main characteristics are summarized in Table 2.

**TABLE 2:** Major CD derivatives and their physicochemical characteristics relevant to encapsulation applications.

CD derivative	Structural modification	Main physicochemical properties	Relevance for encapsulation of active ingredient	References
Hydroxypropyl‐β‐CD (HP‐β‐CD)	Substitution of hydroxyl groups with hydroxypropyl moieties	Markedly increased water solubility; reduced crystallinity; improved guest inclusion capacity	One of the most widely used CD derivatives due to its high aqueous solubility and ability to improve the dispersibility and stability of poorly soluble compounds	Y. Zhang et al. (2021); Ferreira et al. (2023)
Methyl‐β‐CD (M‐β‐CD)	Partial methylation of hydroxyl groups	Enhanced aqueous solubility and increased affinity toward hydrophobic guests	Frequently employed to improve the solubilization and complexation of hydrophobic molecules	Puskás et al. (2023); Matencio et al. (2020)
Sulfobutyl ether‐β‐CD (SBE‐β‐CD)	Introduction of sulfobutyl ether groups	Very high‐water solubility; anionic character; improved complexation efficiency	Suitable for enhancing the aqueous stability and solubility of guest molecules	Ferreira et al. (2023)
Carboxymethyl‐β‐CD (CM‐β‐CD)	Introduction of carboxymethyl groups	Increased water solubility and pH‐responsive behavior	Useful for improving solubility and stability under variable pH conditions	Y. Zhang et al. (2021)
Hydroxypropyl‐γ‐CD (HP‐γ‐CD)	Hydroxypropyl substitution of γ‐CD	High water solubility combined with the larger cavity of γ‐CD	Potentially advantageous for larger guest molecules that cannot be efficiently accommodated by β‐CD	Ferreira et al. (2023)
Branched CDs (e.g., glucosyl‐ and maltosyl‐CDs)	Attachment of oligosaccharide side chains	Improved water solubility and reduced tendency to aggregate	Useful when very high dispersibility in aqueous systems is required	Z. Zhang et al. (2024)

### 1.2 Physicochemical Properties and Solubility Behavior

**FIGURE 1:**
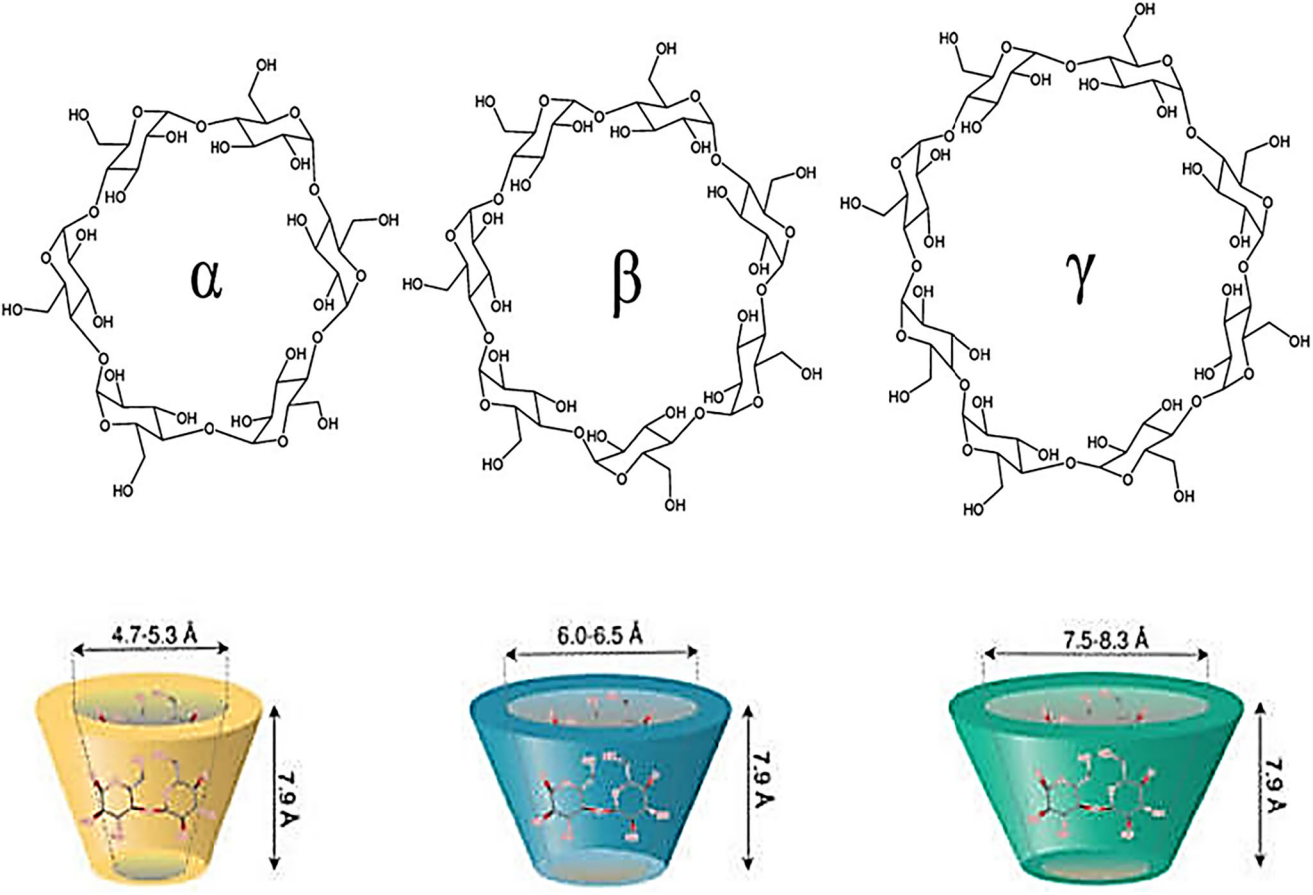
Chemical structures and three‑dimensional architectures of α‑, β‑, and γ‑cyclodextrins (CDs). The upper panel shows the molecular structures of the three CDs, consisting of six, seven, and eight α‑D‑glucopyranose units linked through α‑1,4‑glycosidic bonds. The lower panel depicts their truncated‑cone geometries, highlighting the progressive increase in cavity diameter that underpins differences in inclusion capacity, molecular encapsulation behavior, and host–guest affinity relevant to food matrices. Illustrations rearranged from Poulson et al. (2021) and Saffarionpour (2019).

While the structural differences among the three main types do not affect the height of the internal cavity, they do alter its diameter, which in turn influences certain physicochemical properties (Figure 1; Lachowicz et al. 2020; Poulson et al. 2021).

As shown in Table 1, α‐CD has a smaller cavity and can therefore only encapsulate low molecular weight molecules or compounds with aliphatic side chains, limiting its range of applications. γ‐CD, on the other hand, features a larger cavity but is expensive and difficult to produce on a large scale. The most widely studied and used CD in the food industry is β‐CD, due to its intermediate cavity size and relatively simple and cost‐effective production (Liu et al. 2022).

A key distinction among the three main CDs is their water solubility, which is strongly affected by their structural features: at 25°C, α‐CD has an aqueous solubility of 12.8 g/100 mL, β‐CD is significantly less soluble at 1.8 g/100 mL, while γ‐CD exhibits the highest solubility at 25.6 g/100 mL. This trend persists with rising temperatures: at 45°C, solubility increases to 29.0 g/100 mL for α‐CD, 4.5 g/100 mL for β‐CD, and 58.5 g/100 mL for γ‐CD. At 60°C, the values further rise to 66.2, 9.1, and 129.2 g/100 mL, respectively (Poulson et al. 2021).

The low solubility of β‐CD, compared to the other two forms, is attributable to the strong interactions caused by the formation of intramolecular hydrogen bonds between the secondary hydroxyl groups. These hydrogen bonds create a sort of complete “belt” that limits the ability of β‐CD to form hydrogen bonds with the surrounding water molecules (Wankar et al. 2020).

### 1.3 Inclusion Complexation and Functional Advantages

CDs are capable of forming inclusion complexes with a wide range of compounds of various natures, solid, liquid, or gaseous, through a process known as molecular complexation (Pinho et al. 2014).

This phenomenon relies on a dimensional and physicochemical complementarity between the inner cavity of the CDs, which is lipophilic in nature, and the guest molecule, which becomes incorporated within it. The success of the inclusion process strongly depends on the complementarity between the size and physicochemical properties of the CD (host) and the guest molecule (Astray et al. 2009). Once inside the cavity, the guest molecule undergoes conformational adjustments to optimize van der Waals interactions. The hydrophobic cavity protects lipophilic guest molecules from the surrounding aqueous environment, while the outer surface, rich in polar hydroxyl groups, contributes to the solubilization of the complex (Poulson et al. 2021).

Molecular complexation with CDs is not only simpler and more cost‐effective than most other encapsulation methods but also enhances the physicochemical properties of the guest molecules (Del Valle 2004). Key benefits include improved water solubility, increased stability of labile compounds against degrading agents (such as oxygen, visible and UV light, and heat), reduced volatility and sublimation, and masking unpleasant odors and flavors. In addition, CD encapsulation enables the controlled release of active ingredients or flavor compounds. These advantages make CDs highly versatile and applicable in industries such as food, pharmaceuticals, cosmetics, and textiles (Nicolaescu et al. 2025).

One area where CD‐based encapsulation is seeing growing application is in the stabilization of natural pigments. These compounds, increasingly valued by consumers for their natural origin, often suffer from poor stability when exposed to light, oxygen, heat, and pH changes, as well as from limited water solubility. Many of these limitations can be overcome by using CDs as host molecules, which protect the pigments from degradation while improving their solubility, shelf life, and controlled release capabilities.

### 1.4 Regulatory Framework

Given the increasing interest in CD‐based applications, especially in food, cosmetics, and pharmaceuticals, regulatory frameworks have progressively evolved to ensure their safe use in consumer products. The regulations governing the use of CDs in food vary considerably from one country to another.

In Europe, β‐CD is approved as a food additive (E 459), and the EFSA (European Food Safety Authority) has established an acceptable daily intake (ADI) of 5 mg/kg body weight per day. α‐CD is classified as a novel food, while γ‐CD is subject to limited authorization. This regulatory framework demonstrates a strong focus on risk assessment and consumer protection. In the United States, all three forms of CD have been recognized by the FDA (Food and Drug Administration) as GRAS (Generally Recognized as Safe) and are therefore considered safe for use in food. This classification promotes the immediate and widespread use of CDs in food products, thereby enhancing market competitiveness. However, the greater regulatory flexibility may raise concerns about the accuracy of pre‐market evaluations. In Japan, all CDs are regarded as natural products, and their regulation is primarily based on purity criteria. In Australia and New Zealand, both α‐ and γ‐CD have held novel food status for over 15 years (Gonzalez Pereira et al. 2021).

Current regulatory fragmentation represents a potential barrier to the standardization and industrial application of CD‐encapsulated products. For companies operating on a global scale, this entails the need to adapt formulations and market strategies to different regulatory frameworks. Therefore, greater alignment between regulations would be desirable in order to foster innovation and facilitate market access.

### 1.5 Market Relevance of CDs

In addition to their established regulatory status, CDs continue to attract considerable commercial interest. Recent market analyses estimate the global CD market at approximately $289–301 million, with sustained growth projected over the next decade (Global Market Insights 2024; Adroit Market Research 2024). The pharmaceutical sector currently represents the largest application area, accounting for more than half of total market revenue, reflecting the widespread use of CDs as solubilizing agents, stabilizers, and bioavailability enhancers in drug formulations.

At the same time, the food and beverage sector is expected to experience steady growth, driven by increasing demand for ingredient stabilization, flavor protection, shelf‐life extension, and encapsulation technologies. In this context, CDs are increasingly explored for the delivery and stabilization of natural compounds, including pigments.

Market analyses consistently identify β‐CD as the most widely used and commercially relevant CD derivative, owing to its favorable balance between performance, availability, and cost (Global Market Insights 2024; Adroit Market Research 2024).

### 1.6 Aim and Scope of the Review

In this context, this review aims to provide an up‐to‐date overview of research on the use of CDs for the encapsulation of natural pigments. It highlights the numerous advantages offered by the formation of inclusion complexes, which enhance the stability, solubility, and functional properties of these pigments, substances with great potential but also limitations. Furthermore, the review emphasizes the opportunities that such synergistic interactions offer to the food industry and other sectors, for instance, as an alternative to artificial colorants.

When contrasted with prior state‑of‑the‑art reviews, this work proposes a mechanistic and comparative framework to rationalize CD–pigment interactions across different pigment classes. Specifically, the review integrates structure–function relationships, dominant degradation pathways, and matrix effects to explain why CD encapsulation is highly effective for some pigments (e.g., anthocyanins and xanthophylls) but limited for others (e.g., chlorophylls and carotenes). This approach enables a more predictive interpretation of CD performance beyond case‐by‐case observations, addressing a gap in current literature.

## 2 The Concept of Encapsulation

As previously emphasized, natural pigments constitute a class of bioactive compounds garnering considerable interest owing to their potential health‐promoting properties and application as naturally derived colorants. Nonetheless, their pronounced instability when exposed to adverse environmental conditions markedly constrains their utilization within the food sector. In this regard, encapsulation has emerged as a promising technological approach to surmount these challenges. Encapsulation is a technique developed around 60 years ago with the aim of preserving the stability of bioactive compounds during processing and storage. Widely applied in both the pharmaceutical and food industries to protect bioactive compounds, including pigments, from destabilizing factors such as light, temperature, and humidity, the technique involves enclosing active agents, whether in solid, liquid, or gaseous form, within a stable carrier matrix. This process protects the encapsulated substances and enhances their stability under various environmental conditions, thereby preserving their functionality and enabling controlled release when needed (Klojdovà et al. 2023).

Although encapsulation is often presented as a single technological concept, the performance of different encapsulation methods varies substantially depending on the physicochemical properties of the pigment, the food matrix, and the industrial constraints. For this reason, a critical comparison of encapsulation techniques is essential to better understand their suitability for different classes of natural pigments.

In this review, the focus is placed on the use of CDs; however, various coating materials may be employed (carbohydrate‐, protein‐, and/or lipid‐based carriers) that must be capable of retaining the active ingredients within the complex during both processing and storage, possess suitable rheological properties, and do not react with the encapsulated substance (Soukoulis and Bohn 2018). CD‐carriers represent a particularly promising approach because they already possess many of these characteristics and, through appropriate chemical or physical modifications, can become extremely versatile.

### 2.1 Techniques Applied for Encapsulation

The choice of an encapsulation technique largely depends on the physicochemical properties of the core materials, their intended application, as well as economic and environmental considerations. In light of these factors, encapsulation methods are generally classified into three main categories: physical, physicochemical, and chemical techniques. Each category involves distinct mechanisms and offers specific advantages, making them suitable for a wide range of applications and material types. However, these techniques differ markedly in terms of scalability, cost, suitability for different pigment classes, and technological limitations. Different from prior papers that describe encapsulation techniques independently of pigment chemistry, the present work evaluates each method in relation to pigment‐specific stability constraints, enabling a comparative assessment of their practical suitability. For example, spray‐drying is highly scalable but unsuitable for thermolabile pigments such as chlorophylls and betalains, whereas freeze‐drying offers excellent retention but is economically prohibitive for large‐scale food production (Furuta and Neoh 2021).

#### 2.1.1 Physical Methods

Below are some of the most widely adopted and innovative physical methods, which utilize precise temperature control and, in certain cases, the combined application of low temperatures and reduced pressures to efficiently remove water and produce powders with optimal properties. These techniques primarily rely on physical transformations of the carrier matrix rather than chemical interactions with the bioactive compound to be encapsulated.

##### 2.1.1.1 Spray Drying

Spray drying is a well‐established encapsulation technique that, despite its long‐standing use, continues to be one of the most widely adopted methods in the food and pharmaceutical industries.

Its continued use is attributed to its high encapsulation efficiency, cost‐effectiveness, and the ability to produce stable final products within a relatively short processing time (Assadpour and Jafari 2019). Moreover, this technique offers precise control over the morphology (shape and size) of the resulting powders, which often exhibit controlled release properties (Arpagaus et al. 2018).

Despite its many advantages, this technology carries the risk of irreversibly damaging heat‐sensitive pigments. Temperature, the most critical parameter, if not properly managed, can lead to the degradation of the active compound and significantly affect both yield and final product efficiency (Samborska et al. 2019). Another limitation of this technique is its relatively low yield, typically ranging between 20% and 70%, largely due to powder accumulation on the drying chamber walls during processing (Kandasamy and Naveen 2022).

**FIGURE 2:**
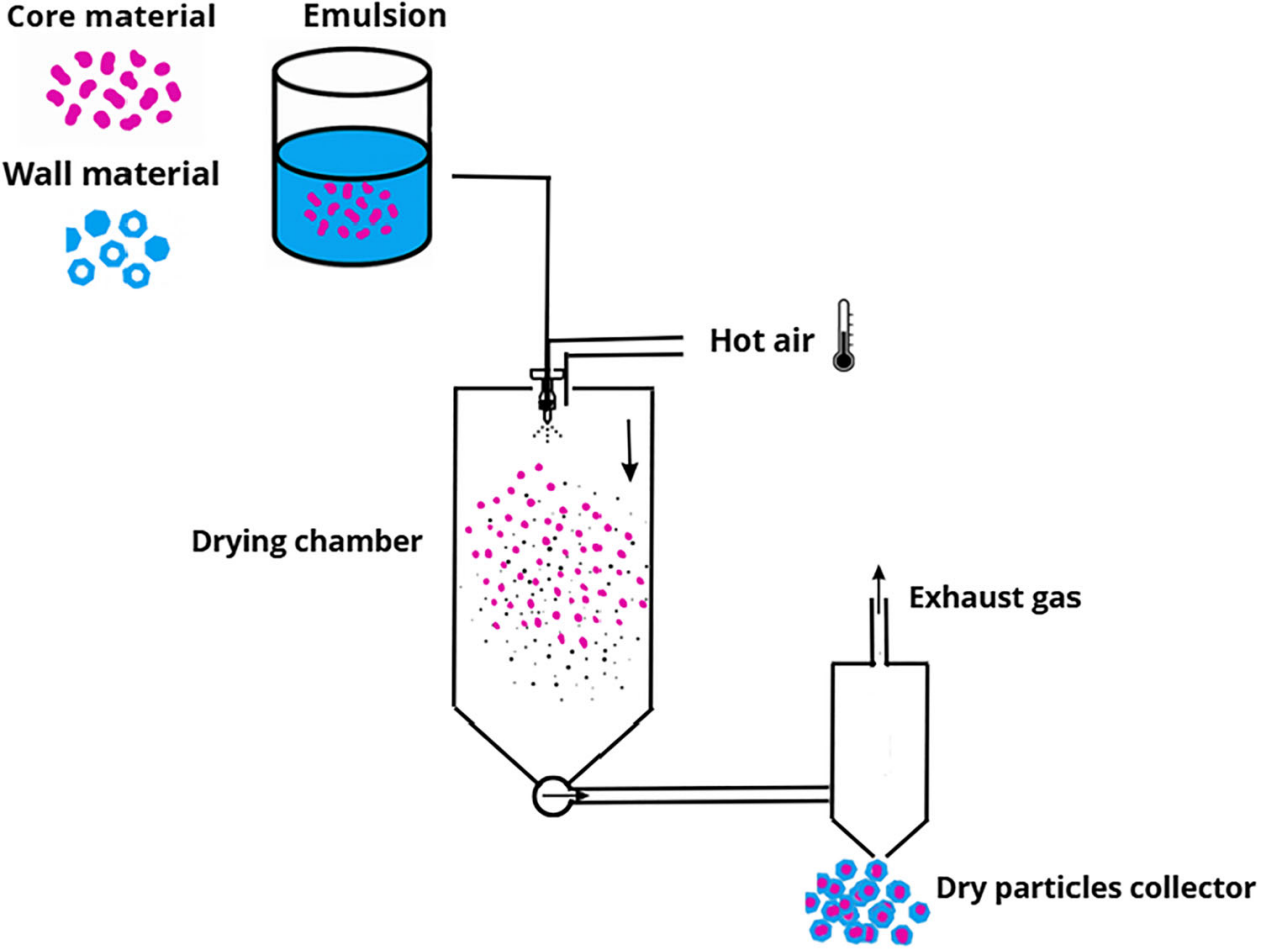
Process flow diagram of spray‐drying encapsulation.

The spray drying technique is employed not only as a dehydration method but, more importantly, for the encapsulation of active compounds. It enables the direct transformation of a fluid into powder through a sequence of steps (Mohammed et al. 2020), as illustrated in Figure 2.

The performance of spray drying for the production of pigment–CD complexes depends strongly on the interplay between formulation and processing parameters. Inlet temperature must be sufficiently high to ensure efficient solvent evaporation and powder formation, but excessive temperatures may promote pigment degradation, particularly in thermolabile compounds such as betalains and chlorophyll derivatives (Díaz‑Montes 2025). Likewise, feed solid content, pigment‐to‐CD ratio, atomization conditions, and feed flow rate influence particle morphology, encapsulation efficiency, moisture content, and pigment retention. Optimized formulations generally require balancing drying efficiency with the preservation of pigment stability, as improved powder recovery may not necessarily correspond to maximum pigment protection. These considerations highlight the importance of jointly optimizing both formulation composition and process parameters when designing spray‐dried CD‐based pigment delivery systems (Díaz‑Montes 2025).

Studies on lipophilic compounds such as α‑tocopherol have demonstrated that β‑CD can improve oxidative stability during spray drying. Do Carmo et al. (2017) reported that the formation of inclusion complexes, particularly when combined with protein‐based carriers such as zein, resulted in powders with regular morphology, good water solubility, and enhanced stability. Nonetheless, the authors also observed that high inlet temperatures still caused measurable degradation of the core material, indicating that the protective effect of the CD cavity is only partial and insufficient to fully counteract thermal stress.

A broader perspective emerges from the systematic review by Díaz‑Montes (2025), which examined the encapsulation of various natural pigments (including carotenoids, anthocyanins, chlorophylls, and betalains) using CDs in spray‑drying processes. The paper highlights that CDs generally enhance thermal and photochemical stability, particularly for carotenoids and anthocyanins. However, for pigments that are intrinsically highly thermolabile, the inclusion complex provides only limited protection. Even when encapsulated, these pigments undergo significant degradation if the drying temperature exceeds their stability threshold, confirming that CDs cannot compensate for excessively harsh processing conditions.

Further insights are provided by other authors, who emphasize that CDs do not form a continuous protective shell around the pigment but rather a host–guest complex in which only part of the molecule is accommodated within the hydrophobic cavity. As a result, the stabilizing effect is inherently partial and strongly influenced by the choice of wall materials, as well as by the drying parameters. The authors also note that the presence of CDs does not substantially improve process yield, as the tendency of powders to adhere to the drying chamber remains largely unchanged (Fuenmayor et al. 2021).

Overall, the literature indicates that spray drying may be an effective approach for producing pigment–CD complexes, but its success depends on a careful balance between formulation and process conditions. CDs can significantly enhance stability against oxidation, light, and pH fluctuations, yet their ability to protect against thermal degradation is limited. For pigments with moderate thermal sensitivity, the technique may yield stable powders with improved functional properties. For highly thermolabile pigments, however, the high temperatures required for efficient drying remain a major constraint, and the use of CDs must be complemented by additional stabilizing strategies or alternative encapsulation methods.

##### 2.1.1.2 Freeze‐Drying

Freeze‐drying, also known as lyophilization, is a three‐step dehydration process that involves the removal of moisture through sublimation. Initially, the homogeneous solution containing the active substance and wall material is frozen at temperatures ranging from –50°C to –80°C, a critical phase during which the compounds of interest may be altered due to ice crystal formation. This is followed by primary drying, where water is removed via sublimation at pressures typically between 0.1 and 0.2 mbar. This phase proceeds slowly and involves the application of heat and, if necessary, partial vacuum. Subsequently, secondary drying removes any unfrozen water molecules by increasing the temperature while maintaining low pressure. At the end of the process, the vacuum is broken with an inert gas, and the final moisture content is reduced to approximately 1%. Freeze‐drying is primarily employed for thermolabile substances and oxidation‐prone food products (Kandasamy and Naveen 2022).

Although this technique yields high‐quality powders, its application in the food industry is limited due to its high operational costs and time‐consuming (Table 3). For instance, the primary drying phase alone typically takes around 12 h (Chhabra et al. 2024). In this context, encapsulation within CDs represents an effective strategy to counteract these processing stresses. These cyclic oligosaccharides form inclusion complexes that shelter sensitive pigments from the thermal and mechanical stresses caused by ice crystal formation (Cid‐Samamed et al. 2022). The hollow structure of the CD shields the guest molecule from oxygen and light, thereby preserving color and significantly improving water solubility. This synergistic approach ensures a highly stable, high‐quality final powder with an extended shelf life (Table 3).

**TABLE 3:** Comparative overview of the principal methods used for the preparation of CD–pigment inclusion complexes.

Method for CD complex preparation	Main advantages	Major limitations	Scalability	Most suitable pigment classes	References
Spray drying	Cost‐effective; rapid processing; high encapsulation efficiency; industrially established; suitable for powder production	Thermal degradation of heat‐sensitive pigments; limited protection against severe thermal stress; powder deposition reduces yield	High	Carotenoids, anthocyanins	Díaz‐Montes (2025)
Freeze‐drying	Excellent retention of thermolabile compounds; minimal thermal degradation; high product quality	High operating costs; long processing times; energy‐intensive	Low–moderate	Betalains, chlorophylls, anthocyanins	Ishwarya et al. (2022)
Spray freeze‐drying (SFD)	Combines low thermal stress with good powder properties; high bioactive retention	Expensive and technologically demanding	Moderate	Thermolabile pigments	Ishwarya et al. (2022)
Fluidized bed granulation	Improves flowability, storage stability, and handling properties	Not a primary encapsulation technique	High	Post‐processing of previously encapsulated pigments	Pawar et al. (2020)
Coacervation	Excellent protection; high encapsulation efficiency; suitable for hydrophilic compounds	Sensitive to pH and ionic strength; complex process control; difficult scale‐up	Low–moderate	Anthocyanins, betalains	Tawiah et al. (2022)
CD inclusion complexes	Mild conditions; low energy consumption; clean‐label potential; improved stability and solubility	Limited by cavity size; less effective for bulky pigments	Moderate	Anthocyanins, betalains, xanthophylls	Gonzalez Pereira et al. (2021); Jug et al. (2025)
Co‐precipitation	Simple and widely used; high complexation efficiency	Solvent use; sometimes lower encapsulation efficiency than kneading	Moderate	Anthocyanins, betalains, curcumin	Jug and Mura (2018); Benucci, Mazzocchi, et al. (2022)
Kneading	Low solvent consumption; suitable for thermolabile pigments; often high encapsulation efficiency	Batch variability; limited industrial scalability	Moderate	Anthocyanins, betalains	Wadhwa et al. (2017); Lobo et al. (2018)
Supercritical CO_2_	Solvent‐free; excellent protection against oxidation; mild temperatures	High equipment costs; specialized operation required	Low–moderate	Carotenoids, curcuminoids	Janiszewska‐Turak (2017); Durante et al. (2016)
Grinding	Green and inexpensive; scalable; solvent‐free	Lower encapsulation efficiency; possible mechanical degradation	High	Crystalline carotenoids	Jug and Mura (2018)
Microwave irradiation	Rapid processing; high encapsulation efficiency	Possible thermal degradation; scale‐up challenges	Moderate	Moderately heat‐stable pigments	Mehta et al. (2022)

In the food and pharmaceutical sectors, encapsulation is primarily performed using the two techniques previously described. These have been subsequently integrated into a single methodology, known as spray freeze‐drying (SFD), which is particularly effective for the encapsulation of volatile and thermolabile compounds. The process consists of three main stages: atomization of the solution, freezing of the droplets in a cryogenic medium, and sublimation of the frozen liquid to yield a dry powder (Adali et al. 2020). This innovative and synergistic technique not only reduces operating costs and processing time but also enhances the retention of bioactive compounds (Dutta et al. 2018).

##### 2.1.1.3 Fluidized bed spray granulation

Although not a true encapsulation technique, the fluidized bed granulator is a widely used physical method for the final formulation of encapsulated products. This technology is mainly employed in the pharmaceutical sector, as it enables the production of granules containing precise and uniformly distributed doses of the active ingredient. It is also used in the food industry, where it improves the rheological properties of powders, leading to significant advantages in storage and transport (Orth et al. 2024; Atxutegi et al. 2024).

The process consists of spraying a solution, suspension, or dispersion containing a binder onto a bed of powders suspended in an air stream. The particles, wetted by the binder, collide and form temporary bonds due to the surface tension of the liquid, leading to the formation of granules.

The quality of the granules depends on several factors, including the initial particle size, the air velocity used for fluidization, and the concentration and flow rate of the binder, as well as other process parameters such as temperature and spraying duration (Pidalà et al. 2025). The integration of fluidized bed spray granulation with CD technology yields a dual‐mechanistic encapsulation framework. At the molecular scale, host–guest inclusion complexation drives the process, driving the lipophilic moieties of the coloring pigments into the hydrophobic cavities of the CD vectors (de Freitas 2019). This architecture isolates the colorant from oxidative and thermal degradation. Concurrently, the macroscopic fluidized bed process builds upon this molecular shielding. Continuous spray application onto fluidized core particles induces rapid solvent evaporation and layer‐by‐layer solid‐state deposition. This synergistic action creates a dense, structured matrix that guarantees uniform pigment distribution within a protective carbohydrate shell (de Freitas 2019). Consequently, this process significantly enhances the chemical stability, dissolution kinetics, and industrial flowability of the target granulate, outperforming conventional spray‐drying techniques (Table 3).

#### 2.1.2 Physicochemical Methods

##### 2.1.2.1 Coacervation

The first stage consists of preparing the dispersion or emulsion: the pigment, or any other material, is dispersed in a cationic aqueous polymer, to which a second solution is added, usually containing an anionic polymer. This is followed by the encapsulation phase, during which the complex forms upon agitation, as the wall material deposits onto the particles to be encapsulated. The process is facilitated by the addition of salt and/or changes in pH and temperature. Finally, the encapsulated material is stabilized. At the end, the microcapsules are separated and dried by spray drying or freeze‐drying, resulting in the final product (Tawiah et al. 2022). The integration of CD inclusion complexation with coacervation represents a powerful synergistic approach for the encapsulation of sensitive coloring pigments. In this dual‐layered delivery system, the pigment is first accommodated within the hydrophobic cavity of the CD host, forming a stable guest–host inclusion complex (Fuenmayor et al. 2021). This primary molecular shielding enhances the water solubility of the pigment and protects its core chromophore from chemical degradation. Subsequently, these CD–pigment complexes are subjected to coacervation, where they are trapped within a secondary macromolecular matrix formed by the phase separation of oppositely charged polymers. This dual‐functional architecture provides an advanced barrier that significantly reduces core leaching and improves resistance against environmental stressors such as heat, light, and oxidation (Fuenmayor et al. 2021). Consequently, combining these two techniques offers a highly effective strategy for extending the shelf life and maintaining the visual appeal of natural colorants in complex food, cosmetic, and pharmaceutical matrices.

#### 2.1.3 Chemical Methods

##### 2.1.3.1 Inclusion complexes

Inclusion complexation is an encapsulation technique that involves trapping an active compound (ligand) within the cavity of a host molecule (substrate) through non‐covalent interactions, such as hydrogen bonding, van der Waals forces, or hydrophobic effects driven by entropy. In the food industry, this technology is primarily used to protect and stabilize volatile or low‐molecular‐weight compounds. However, the range of materials capable of forming such complexes is limited. Among the most widely used are β‐CD and β‐lactoglobulin, which offer good encapsulation capabilities and significantly enhance the stability of the active ingredients (Fuenmayor et al. 2021).

**FIGURE 3:**
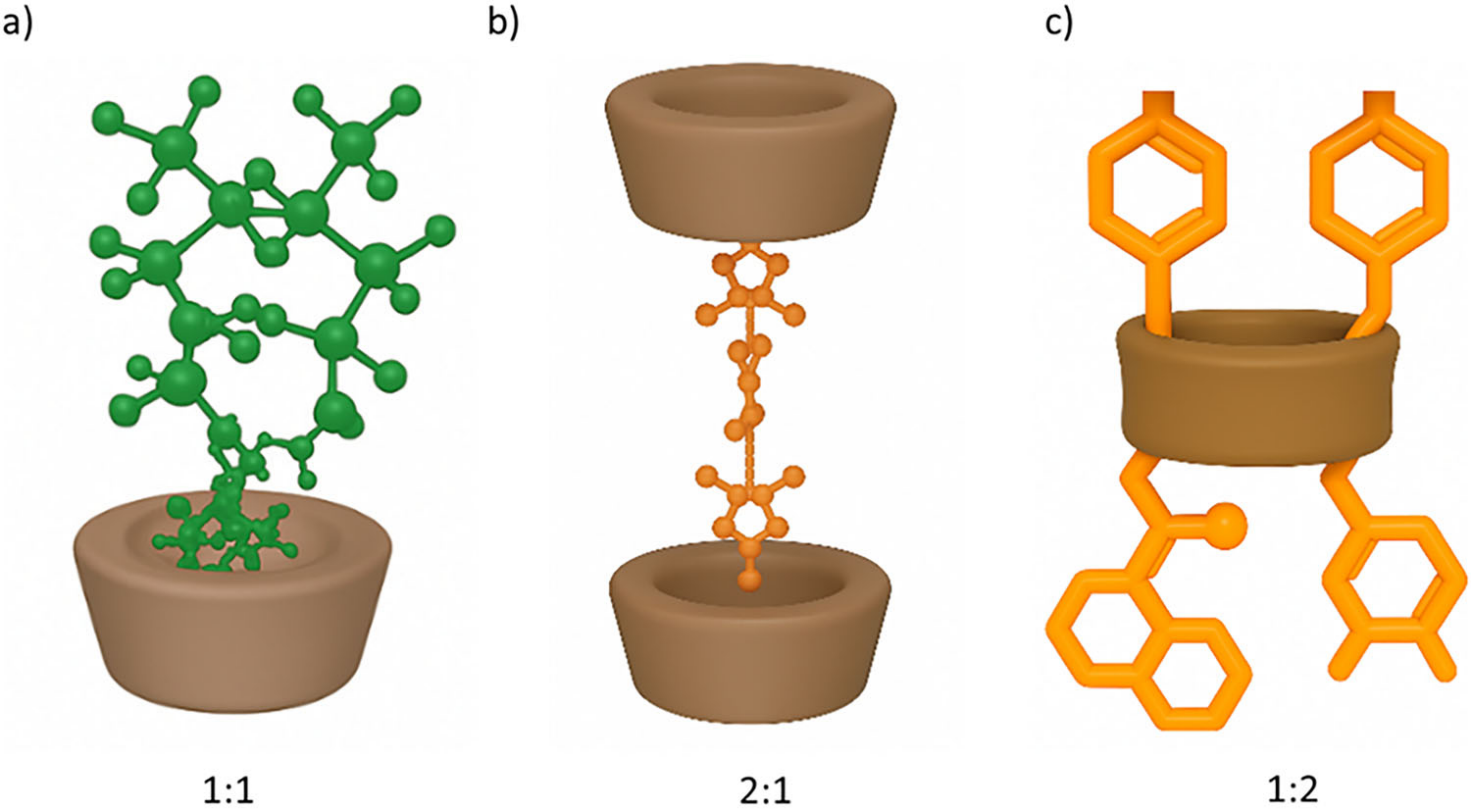
CD–pigment inclusion complexes with different stoichiometric ratios. (6a) β‐CD/chlorophyll at a 1:1 ratio; (6b) β‐CD/curcumin at a 2:1 ratio; (6c) β‐CD/curcumin at a 1:2 ratio.

Depending on the number of host and guest molecules involved, different inclusion ratios can occur. The most common is the 1:1 ratio, known as molecular encapsulation, in which a single encapsulating molecule forms a complex with one guest molecule, as observed for chlorophyll (Lombardelli, Benucci, Pippolini, and Esti 2025; Figure 3a). In some cases, a 2:1 ratio may occur, where two encapsulating molecules encapsulate a single guest molecule, as observed for curcumin (Benucci, Mazzocchi, et al. 2022; Figure 3b). Conversely, for the same pigment (curcumin) a different ratio can be found: 1:2 ratio, one encapsulating molecule may accommodate two guest molecules within its structure (Lobo et al. 2018; Cid‐Samamed et al. 2022; Figure 3c). A 2:2 ratio can also occur, involving the interaction of two encapsulating molecules with two guest molecules, although this is less common. To the best of our knowledge, and based on the literature surveyed for this review, no examples of natural pigments forming 2:2 inclusion complexes with CDs have been explicitly reported. Most studies have instead described alternative stoichiometries, depending on the structural characteristics of both the pigment and the CD (Fuenmayor et al. 2021).

There are several methods available for the preparation of inclusion complexes with CDs. The most commonly used techniques include co‐precipitation, kneading**, **supercritical carbon dioxide processing, grinding, microwave irradiation, and spray‐drying, among others.

##### 2.1.3.2 Co‐Precipitation Method

This technique involves adding, under agitation, an organic solution (e.g., ethanol‐based) containing the compound to be encapsulated (guest) into an aqueous solution where CD is dissolved. Upon subsequent cooling of the mixture, crystallization and precipitation of the inclusion complex occur. Unencapsulated guest compounds are then removed by washing. Co‐precipitation is considered one of the most widely used methods for forming complexes with CDs, thanks to its operational simplicity and high efficiency (Jiang et al. 2019).

Nonetheless, under specific conditions, this method may exhibit lower encapsulation efficiency compared to simpler techniques. Benucci, Mazzocchi, et al. (2022), for example, reported an encapsulation efficiency of 95% for β‐CD/curcumin complexes prepared via simple mixing, whereas the same complexes obtained through co‐precipitation achieved only 72%. Despite this discrepancy, no significant differences were observed between the two methods in terms of loading capacity or color strength (Table 3).

##### 2.1.3.3 Kneading Method

This is a simple technique that involves the formation of a paste by mixing CDs with water.

The host compound is subsequently incorporated into this semi‐solid matrix through homogenization (da Silva Júnior et al. 2017). The resulting solid is then washed with a small amount of solvent to remove any uncomplexed material (Wadhwa et al. 2017).

Despite its operational simplicity, this method has demonstrated superior performance, compared to co‐precipitation, particularly in the protection of pigments encapsulated in isotonic beverages, as reported by Lobo et al. (2018).

##### 2.1.3.4 Supercritical Carbon Dioxide Processing

The formation of the inclusion complex with CDs using supercritical CO_2_ occurs through several stages. Initially, the CD and the guest compound are placed in a thermostatically controlled and pressurized autoclave, into which supercritical carbon dioxide is then introduced. Thanks to its unique properties, the supercritical CO_2_ acts as a “green” solvent, promoting the dissolution of the guest compound and improving its contact with CD, thereby facilitating the formation of the inclusion complex. Once the complex has formed, the autoclave is depressurized, causing the CO_2_ to vaporize and resulting in the separation and precipitation of the complex in solid form.

The main advantage of this technique is the absence of solvent residues since the carbon dioxide evaporates completely, ensuring higher purity and safety of the final product (Banchero 2021).

##### 2.1.3.5 Grinding Method

This approach consists in the mechanical grinding of a solid mixture consisting of the compound of interest (guest) and CD. The mechanical action induces crystal breakage, resulting in a reduction in particle size and an increase in the contact surface between the two components, thus favoring the interaction and formation of the inclusion complex. It is a simple, economical and environmentally friendly technique since it does not require the use of solvents, being among the “green” methods for the preparation of CD‐based complexes (Jug and Mura 2018).

##### 2.1.3.6 Microwave Irradiation

The microwave irradiation method for the preparation of CD inclusion complexes begins with the formation of a homogeneous paste, obtained by mixing the guest compound with CD in defined proportions. A small amount of an aqueous or hydroalcoholic solution is then added to the solid mixture to achieve a workable, paste‐like consistency.

The resulting paste is then subjected to microwave treatment under controlled power and time conditions. This process enables rapid and uniform heating, which promotes the interaction between the CD and the guest molecule, thereby facilitating the formation of the inclusion complex.

Following microwave irradiation, the material is dried in an oven at moderate temperature (typically around 50°C) to remove any residual solvent and stabilize the resulting complex.

Finally, once dried, the product is pulverized and sieved to obtain a fine, uniform powder suitable for further application (Patel et al. 2024).

As summarized in Table 3, each encapsulation method presents specific advantages and limitations depending on pigment chemistry, processing requirements, and economic constraints. Consequently, the selection of an appropriate technique should balance encapsulation efficiency, pigment stability, scalability and industrial feasibility (Alu'datt et al. 2022).

### 2.2 Analytical Techniques for Inclusion Complex Characterization

The characterization of guest‐CD systems and the confirmation of inclusion complex formation require the use of various analytical techniques. Analysis of the obtained results provides insights into the interactions between the components, thereby supporting the selection of the most suitable CD for a given host molecule, as well as the identification of the most appropriate preparation method and experimental conditions (Cid‐Samamed et al. 2022).

The analytical techniques used to characterize CD complexes vary depending on their physical state. When the complexes are in the solid state, techniques such as scanning electron microscopy (SEM), Fourier transform infrared spectroscopy (FTIR), powder X‐ray diffraction (PXRD), Raman spectroscopy, thermogravimetric analysis (TGA), and differential scanning calorimetry (DSC) are employed. In contrast, for complexes in solution, different methods are used, including UV‐visible spectroscopy, nuclear magnetic resonance (NMR), fluorescence spectroscopy, circular dichroism, and high‐performance liquid chromatography (HPLC; Kfoury et al. 2018; Sarabia‐Vallejo et al. 2023). Table 4 provides a brief description of the techniques used to characterize host–CD inclusion complexes. Beyond confirming complex formation, the characterization of natural pigment delivery systems should also evaluate the preservation of color attributes, which ultimately determine their technological relevance in food application. For this reason, colorimetric analysis is frequently employed alongside spectroscopic and thermal techniques to evaluate the effectiveness of CD encapsulation. Measurements based on the CIELAB color space (L*, a*, b*) and the corresponding total color difference (ΔE*) provide quantitative information on color retention during processing and storage (Lombardelli, Benucci, Pippolini, Marabottini, and Esti 2025). This aspect is particularly relevant for anthocyanins and betalains, whose degradation is often accompanied by pronounced changes in redness (a*) and overall color intensity, as well as for carotenoids, where oxidation and isomerization can lead to reduction in yellowness (b*) and color saturation (Gomes et al. 2014; Lobo et al. 2018). Consequently, colorimetric parameters represent not only quality indicators but also indirect markers of pigment stability, complementing chemical analyses such as HPLC and UV–Vis spectroscopy. The combined use of color and compositional analyses is therefore recommended when evaluating the performance of CD‐based delivery systems for natural pigments in real food matrices.

**TABLE 4:** Summary of analytical techniques commonly employed for the characterization of host–CD inclusion complexes.

Technique	Application phase	Description	References
Scanning electron microscopy (SEM)	Solid	It enables the examination of the surface morphology of the samples. It reveals structural modifications that suggest interactions between the components of the complex.	Mura (2015), Espenti et al. (2025)
Fourier‐transform infrared spectroscopy (FT‐IR)	Solid	Technique based on the analysis of molecular vibrations. Changes in the characteristic bands of the guest molecule suggest complex formation with the CD. It is low cost, sensitive, selective, and allows easy spectral acquisition. However, it requires complex sample preparation (KBr powder) with potential sample alteration due to the hygroscopic nature of KBr.	Mura (2015)
Powder X‐ray diffraction (PXRD)	Solid	PXRD rapidly identifies crystalline phases and assesses crystallinity without sample preparation or alteration, allowing reuse.	Mura (2015)
Raman spectroscopy	Solid	Molecular technique based on the inelastic scattering of light, useful for identifying the vibrational states of molecules and monitoring changes in the structure of chemical bonds.	Mura (2015)
Thermogravimetric analysis (TGA)	Solid	Measures sample mass changes during controlled heating; thermograms show thermal decomposition. Inclusion complexes show altered thermograms due to increased thermal stability of the guest molecule.	Saadatkhah et al. (2020)
Differential scanning calorimetry (DSC)	Solid	Thermal technique that measures heat flow during phase transitions. Complex formation with β‐CD can be indicated by shifts or disappearance of the guest's melting peaks.	Almoselhy (2020)
Ultraviolet/visible (UV‐Vis) spectroscopy	Solution	UV‐Vis spectroscopy detects changes in absorption spectra that indicate complex formation; it allows the determination of formation constants and stoichiometry.	Lombardelli, Benucci, Pippolini, and Esti (2025); Jin et al. (2020)
Nuclear magnetic resonance (NMR) spectroscopy	Solution	NMR spectroscopy provides detailed structural information on CD inclusion complexes, revealing host orientation, conformation, and intermolecular interactions, making it essential for complex characterization.	Mura (2014)
Fluorescence spectroscopy	Solution	It is a fast, sensitive method for studying CD complexes with fluorescent guests, showing increased fluorescence upon encapsulation; however, it is limited to fluorescent compounds and requires careful sample preparation.	Kfoury et al. (2018)
Circular dichroism spectroscopy	Solution	Technique for determining pure chiral compounds, similar to UV‐Vis spectroscopy. It's mainly used for the structural analysis of proteins; however, being expensive, the technique is not very widely applied.	Kraszni et al. (2023)
High‐performance liquid chromatography (HPLC)	Solution	HPLC operates on the principle of separating the components of a mixture based on their interactions with the stationary and mobile phase.	Hussein (2025)

## 3 Encapsulation of Natural Pigments in CDs

### 3.1 Natural Pigments: Characteristics, Limitations, and Potential

Color is one of the first sensory attributes that captures consumer attention, making it a key factor in the food industry. For this reason, food products are often enhanced with colorants. Among these, synthetic azo‐compounds are the most widely used due to their high water‐solubility and chemical stability (Fuenmayor et al. 2021).

Despite the fact that adverse effects of most synthetic food colorants have been observed only at high doses and after long‐term exposure in conventional toxicity tests, growing consumer concern and skepticism about their safety have led to increased scientific interest in natural food colorants (Amchova et al. 2024).

Natural food colorants, also known as organic pigments, are secondary metabolites derived from vegetables, fruits, plants, minerals, and other edible natural sources. These compounds play numerous functional roles throughout the plant life cycle and are also believed to offer potential health benefits when consumed. However, natural dyes, despite their promising pigmenting potential, present several limitations when applied in food system (Ghosh et al. 2022). They are typically more expensive, may not reproduce the exact hues of synthetic counterparts, can impart undesirable flavors or aromas, and often lack stability. Such instability arises because, once extracted from their natural sources, these pigments are exposed to new physicochemical conditions (e.g., pH, temperature, light) that remarkably differ from their native environment (Malabadi et al. 2022; Das and Maulik 2024).

**FIGURE 4:**
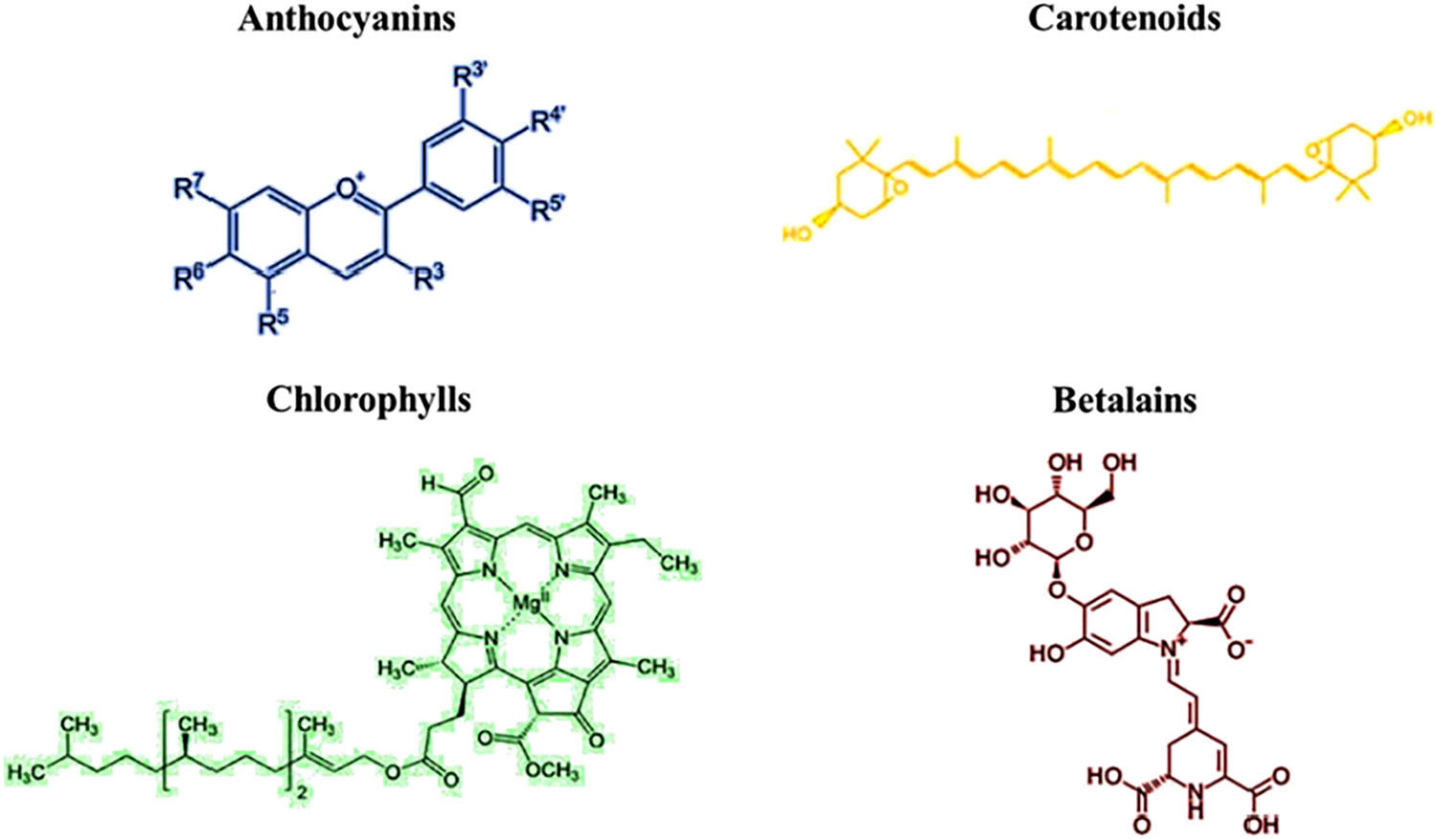
Chemical structure of major classes of natural pigments (Benucci, Lombardelli, et al. 2022).

**FIGURE 5:**
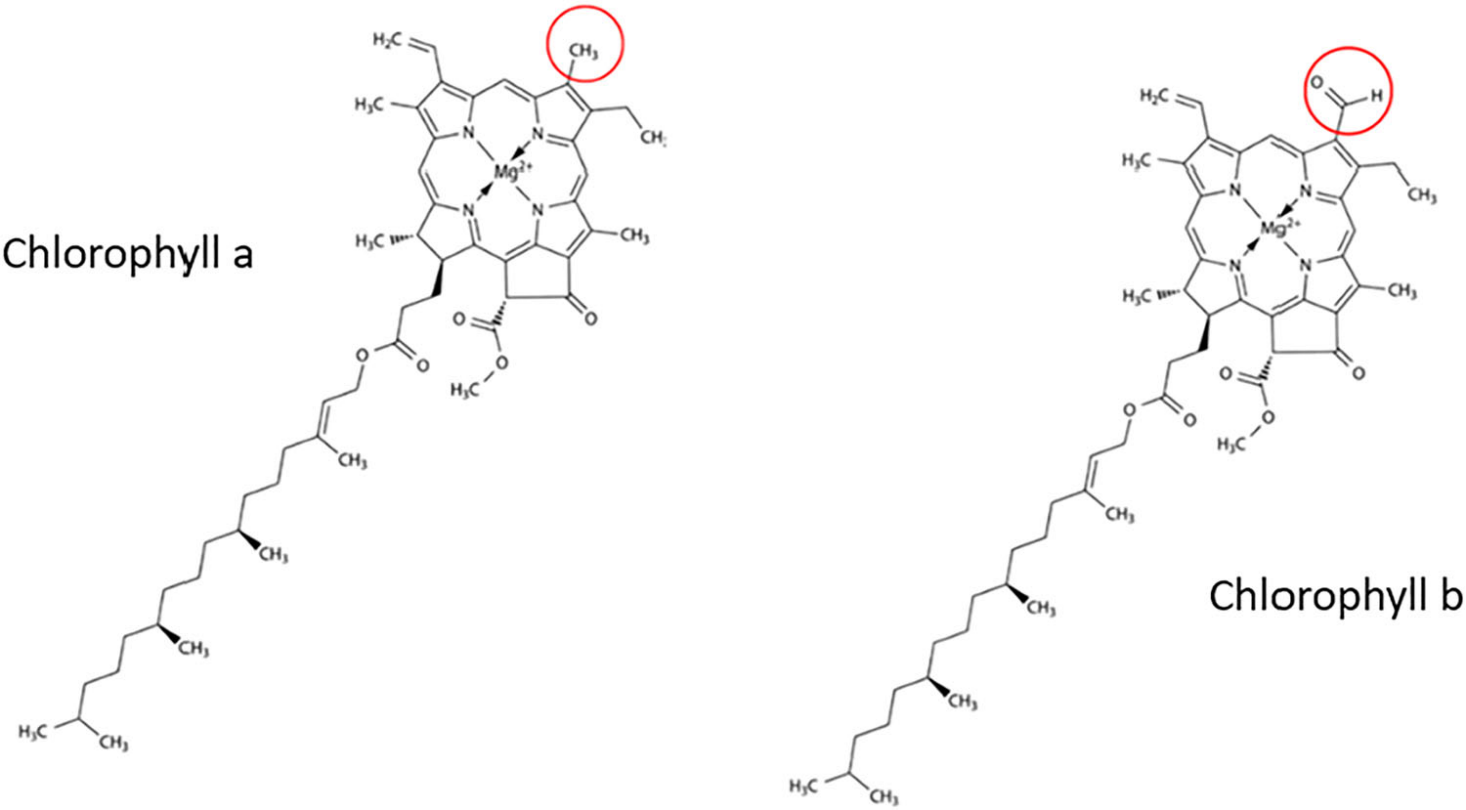
Chemical structure of chlorophyll a and chlorophyll b, with the structural difference highlighted in red.

The main classes of pigments derived from plants can be grouped into four primary categories: carotenoids, chlorophylls, flavonoids (anthocyanins), and betalains (Figures 4 and 5).

#### 3.1.1 Carotenoids

Among natural pigments, carotenoids are the most widespread lipophilic colorants and among the most extensively studied. They are widely distributed in plants, fruits, vegetables, algae, and photosynthetic microorganisms, where they are responsible for yellow, orange, and red coloration (Joshi et al. 2023). Chemically, carotenoids are C40 tetraterpenes classified into carotenes, which are non‐oxygenated hydrocarbons, and xanthophylls, which contain oxygenated functional groups (Badmus et al. 2022; Wahyuni et al. 2020). Owing to their coloring properties and health‐promoting effects, including antioxidant activity, carotenoids are widely used as dietary supplements and natural food colorants (Soukolis and Bohn 2018; Ashokkumar et al. 2023; Bufka et al. 2024).

However, their application in aqueous food systems is often limited by poor water solubility and low physicochemical stability (Fuenmayor et al. 2021). The highly conjugated polyene structure that confers color and bioactivity also makes carotenoids particularly susceptible to oxidation, isomerization, and other degradation reactions triggered by light, oxygen, heat, extreme pH conditions, humidity, and transition metals (Thakur et al. 2017; Ribeiro et al. 2018; Moreno et al. 2021; Miranda et al. 2021; Kaur et al. 2021; Pradhan et al. 2021). These limitations have stimulated extensive research into CD‐based delivery systems. Nevertheless, the elongated structure of many carotenoids is often poorly matched to the relatively small cavities of α‐, β‐, and γ‐CDs, resulting in only partial inclusion (Fuenmayor et al. 2021). The poor match with these standard CDs has shifted much research toward chemically modified CDs. For example, HP‐β‐CD and larger CD‐based nanosponges often provide better steric accommodation for these elongated bioactive pigments (Celitan et al. 2021).

Molecular docking simulations and thermodynamic analyses have elucidated that the structural basis of carotenoid–CD inclusion complexes is primarily driven by the hydrophobic effect, which triggers the displacement of high‐energy water molecules from the host cavity (Clercq et al. 2021). Specifically, the highly conjugated, linear polyene backbone of the carotenoid molecule acts as the primary guest segment, threading directly into the apolar inner cavity of the CD truncated cone (Z. Zhang et al. 2024). Computational modeling reveals that while the rigid central chain resides within the hydrophobic pocket, the bulky terminal ionone rings are strategically positioned. In the case of xanthophylls such as lutein complexed with native β‐CD, these rings anchor at the wider secondary hydroxyl rim (F. Zhang et al. 2024). Conversely, for keto‐carotenoids like astaxanthin encapsulated within modified derivatives such as HP‐β‐CD, the terminal functional groups establish an interlocking intermolecular hydrogen bonding network with the outer hydroxyl groups of the host. This specific spatial arrangement and conformational restriction dramatically enhance the functional stability of the pigment (Wulandari et al. 2023). The physical shielding provided by the CD shell isolates the fragile carbon–carbon double bonds (C = C) from external reactive oxygen species, singlet oxygen, and free radicals, thereby minimizing photo‐oxidative degradation. Furthermore, the steric confinement within the tight cavity limits the rotational freedom of the polyene backbone, effectively suppressing thermally or photochemically induced trans‐to‐cis isomerization pathways (Jug et al. 2025).

Bioavailability constitutes a relevant and often limiting aspect in the use of natural pigments; Xu et al. (2021) showed that the encapsulation of lutein with stevioside and β‐CD by spray drying doubled its in vitro bioavailability, compared to the free compound. By improving the bioavailability of natural pigments through encapsulation, it is possible to enhance their absorption and beneficial effects, as well as their coloring power in food. However, although these results are promising, they are based on in vitro studies and therefore require further in vivo confirmation.

Recent studies have explored the potential of CDs to improve the dispersibility and functional performance of carotenoids in food systems. β‐Carotene inclusion complexes with modified β‐CD have been successfully employed to stabilize Pickering emulsions, enhancing aqueous dispersion and reducing degradation induced by light and oxidation (Niu et al. 2019). In addition, α‐CD has been used to encapsulate carotenoid‐rich oleoresins, producing stable powders with improved handling properties and oxidative stability (Durante et al. 2016). Collectively, these studies demonstrate that CD‐based systems can improve the technological functionality of lipophilic carotenoids and facilitate their incorporation into food formulations.

However, results obtained in real food matrices remain variable. Studies in yogurt and beverage systems consistently report improved dispersibility, color uniformity, and reduced precipitation, whereas protection against oxidation and thermal degradation is often only partial and strongly affected by matrix composition (Gomes et al. 2014; Lobo et al. 2018). Likewise, more complex formulations combining CDs with proteins or polysaccharides may further enhance solubility, bioaccessibility, and thermal stability, although their industrial scalability remains uncertain (Lakshmanan et al. 2022). Overall, the available evidence indicates that CDs are effective tools for improving the functionality and short‐term stability of carotenoids, but they do not completely prevent oxidation, isomerization, or degradation under severe processing conditions. Future research should focus on long‐term stability under realistic storage conditions, comparisons with alternative encapsulation technologies, and a better understanding of interactions between carotenoids, CDs, and food matrix components (Kfoury et al. 2018; Focsan et al. 2019).

Recent advances in functional food science have increasingly explored ternary delivery systems combining CDs with proteins or polysaccharides to address the poor water solubility and limited stability of carotenoids. In these systems, the carotenoid is first complexed within the hydrophobic cavity of a CD, typically β‐CD or HP‐β‐CD and subsequently incorporated into a secondary biopolymeric matrix. Such an approach aims to combine the molecular encapsulation capacity of CDs with the structural and protective functions of biopolymers (Lakshmanan et al. 2022; Celitan et al. 2022).

Evidence from the literature suggests that the addition of a biopolymeric component may provide benefits beyond those achievable with binary CD complexes alone. For example, β‐carotene/β‐CD/chitosan systems have been reported to exhibit improved photostability and enhanced rheological properties owing to hydrogen‐bonding interactions between chitosan and CD molecules, making them attractive candidates for emerging applications such as 3D food printing (H. Wang et al. 2022). Likewise, ternary systems prepared by spray drying using HP‐β‐CD, carrageenan, and soy protein isolate achieved encapsulation efficiencies exceeding 84% and displayed high colloidal stability, as indicated by zeta potential values close to −45 mV (Lakshmanan et al. 2022).

Importantly, the incorporation of proteins and polysaccharides can improve gastrointestinal protection and carotenoid bioaccessibility. Lakshmanan et al. (2022) reported that the in vitro bioaccessibility of β‐carotene increased from approximately 34% in binary β‐carotene–HP‐β‐CD complexes to 78% in the corresponding ternary HP‐β‐CD/carrageenan/soy protein system. Similarly, Celitan et al. (2022) demonstrated that coating β‐carotene–HP‐β‐CD complexes with pectin significantly improved thermal and UV stability compared with the binary formulation. These findings indicate that ternary systems may provide synergistic advantages through the combined effects of CD inclusion complexation and biopolymer‐based physical protection. Nevertheless, additional studies comparing binary and ternary formulations under identical processing and storage conditions are still required to establish whether the technological benefits consistently justify the increased formulation complexity and production costs.

Further investigations into other pigments are reported in Table 5, offering a more comprehensive perspective on the current literature.

**TABLE 5:** Examples of natural pigment encapsulation with CDs—Comparison of techniques, types of CDs, and benefits achieved.

Pigment	Encapsulation technique	Type of CD	Main advantages obtained	Reference
Chlorophyll from spinach	Inclusion complexes (simple mixing)	β‐CD	• Enhanced solubility • Greater stability at pH (3–7) • Greater stability and better color retention over time, even under critical conditions (40°C, UV light exposure)	Lombardelli, Benucci, Pippolini, and Esti (2025)
Chlorophyll from spinach	Inclusion complexes (simple mixing)	β‐CD	• Successful application in food: the drink colored with the encapsulated pigment showed significantly less color change, compared to the one colored with the free pigment	Lombardelli, Benucci, Pippolini, Marabottini, and Esti (2025)
Anthocyanins from strawberry	Inclusion complexes	β‐CD	Greater resistance to H_2_O_2_‐induced oxidation Improved stability under natural light and UV radiation	Ali et al. (2024)
Curcumin	Inclusion complexes (co‐precipitation and simple mixing)	β‐CD	Enhanced water solubility (20% higher than pure dye) Improved stability under different conditions (high temperature and exposure to light)	Benucci, Mazzocchi, et al. (2022)
Lutein (carotenoid)	Spray drying	β‐CD	Double bioavailability compared to free or simple mixture of lutein	Xu et al. (2021)
Natural dyes extracted from various botanical sources	Post hoc encapsulation/retrospective encapsulation	β−CD	• Increased color stability on silk	Salimpour and Malek (2021)
Monascorubrin (red‐orange pigment produced by fungi of the genus *Monascus)*	Inclusion complexes (freeze‐drying method)	HP‐β‐CD	• Increased water solubility • Color maintenance in the pH range 3.5–9	Wu et al. (2020)
Carotenoids from yellow pepper	Inclusion complexes (ultrasound and kneading method)	β‐CD	Greater long‐term stability of the complex obtained with the kneading method Successful application in food: the beverage colored with encapsulated pigment showed minimal color changes during storage	Lobo et al. (2018)
Curcumin	Inclusion complexes (co‐precipitation, freeze‐drying, and solvent evaporation)	β‐CD	Increased water solubility Inclusion complexes using co‐precipitation: most effective technique, enhanced stability against external factors; good results for coloring in ice cream.	Mangolim et al. (2014)
Carotenoids from red pepper	Inclusion complexes (magnetic‐stirring and ultrasonic homogenization)	β‐CD	Promising encapsulation efficiency, particularly with ultrasonic homogenization Increased color stability, tested in yogurt, comparable to the artificial colorant FD&C No. 6	Gomes et al. (2014)
Curcumin	Inclusion complexes (solvent‐evaporation, freeze‐drying, pH shift)	HP‐β‐CD	High curcumin content and improved stability in the inclusion complexes obtained by solvent evaporation and freeze‐drying methods (> 88.39%) Significant increase in curcumin solubility with all methods (> 276.43‐fold increase)	Jantarat et al. (2014)
Curcumin	Grinding method		Increased water solubility of curcumin (more than 20‐fold) with increasing concentrations of HP‐β‐CD Improved light stability after 10 days at 4500 lx Residual curcumin: 45.33% (free form) vs. 73.57% (HP‐β‐CD complexes)	Sun et al. (2014)
Betalains from pitaya peel	Inclusion complexes (co‐precipitation)	β‐CD	Increased water solubility	Mimi Sakinah et al. (2009)
Bixin from *urucum* seeds (carotenoids)	Inclusion complexes (co‐precipitation)	α‐CD	Protection against various stress factors: air, ozone, light and temperature Oxygen protection after 6 days Color loss 26% (free bixin) vs. 1.4% (complexed) Protection against light Degradation: 27% (free bixin) vs. 1.75% (complexed) Protection against light and oxygen after 6 days Degradation: 45% (free bixin) vs. 15% (complexed) Protection against ozone Degradation: 69%–79% (free bixin) vs. 27%–37% (complexed) Thermal protection limited to 50°C only	Lyng et al. (2005)
High‐carotenoid canola Oil	Inclusion complexes	α‐CD e β‐CD	Carotenoid stability (27 days, dark bottles, room temperature)	Basu and Vecchio (2001)

#### 3.1.2 Chlorophylls

Chlorophylls are essential photosynthetic plant pigments responsible for the green color of fruits and vegetables and widely distributed in nature (Humphrey 2004).

Chemically, they belong to the porphyrin family, characterized by a tetrapyrrolic ring system coordinated to a central magnesium (Mg) atom, and an attached lipophilic phytol chain that promotes membrane association (Ebrahimi et al. 2023). Due to their strongly lipophilic nature, chlorophylls are practically insoluble in water, influencing their biological role and industrial extraction (Lefebvre et al. 2020).

Although several forms exist, chlorophyll a and chlorophyll b are the main types in higher plants, localized in chloroplasts and the only forms used in industrial applications (Nabi et al. 2023). Chlorophyll a is the primary light‐absorbing pigment in photosynthesis and is used as a natural colorant in food and pharmaceutical industries. Chlorophyll b differs by a methyl‐to‐aldehyde substitution (–CH_3_ → –CHO), resulting in different optical properties: blue‐green for chlorophyll a and yellow‐green for chlorophyll b, typically present in a 3:1 ratio in higher plants (Carrillo et al. 2021).

Chlorophylls are also associated with nutritional and bioactive properties, being sources of vitamins (E, A, C, K), essential fatty acids and minerals such as magnesium and iron. They exhibit antioxidant, anticancer, antimicrobial, anti‐inflammatory, neuroprotective, and antimutagenic activities (Y. Li et al. 2019) and may help protect against endocrine disruptors (Martins et al. 2023; Ahmadi et al. 2022).

Chlorophyll is a highly unstable pigment that degrades through enzymes such as chlorophyllase, Mg‐dechelatase, pheophytinase, peroxidase, and chlorophyll oxidase (An et al. 2022; Tanaka and Ito 2025).

Environmental factors further accelerate degradation, leading to structural modifications that affect color and nutritional value. When the central Mg^2^^+^ is replaced by two protons (H^+^), chlorophyll converts into derivatives such as pheophytins, pyropheophytins, chlorophyllides, and pheophorbides, causing a color shift from bright green to yellow‐brown (Amin et al. 2021).

Key factors influencing stability include temperature, pH, and light. Chlorophyll is highly thermolabile, more sensitive to heat than carotenoids. pH strongly affects stability, with significant degradation of commercial chlorophyll E141(i) observed at pH 3 and pH 7, compared to intermediate values (Lombardelli, Benucci, Pippolini, and Esti 2025). Finally, UV light, especially combined with high temperature, represents another major destabilizing factor (Lombardelli, Benucci, Pippolini, and Esti 2025).

Although the bulky porphyrin macrocycle of chlorophylls generally prevents true inclusion within the CD cavity, some studies suggest that partial insertion of specific molecular fragments or the formation of external, non‐inclusion associations may still confer measurable stabilizing effects under selected conditions. This indicates that, while full host–guest encapsulation is unlikely, CDs can nonetheless interact with chlorophylls in ways that merit further investigation, particularly for modified derivatives or tailored CD structures (Kelanne et al. 2024).

A relevant contribution to the understanding of chlorophyll–CD interactions was reported by Semeraro et al. (2018), who investigated supramolecular complexes between chlorophyll a and several modified CDs, including HP‐β‐CD, HP‐γ‐CD, DIMEB, and TRIMEB. The study showed that CDs significantly increased the apparent solubility of chlorophyll a in aqueous media and reduced pigment aggregation, maintaining the molecule predominantly in its monomeric form. This effect is attributed to host–guest interactions involving the hydrophobic regions of chlorophyll, particularly the phytol chain, which limits pigment–pigment interactions and enhances dispersion stability.

In a recent study (Table 5) on a green isotonic beverage, β‐CD complexation of commercial chlorophyll E141(i) improved color retention during storage and reduced the rate of browning. However, degradation still progressed significantly, especially under light exposure, indicating that CDs can delay but not prevent the loss of the central magnesium ion and the formation of pheophytins (Lombardelli, Benucci, Pippolini, and Esti 2025). Similar conclusions emerge from the work on β‐CD complexes with natural chlorophyll extracted via enzyme‐assisted processes and with commercial copper‐chlorophyllin: Although β‐CD improved pigment dispersibility and reduced precipitation, the complexes provided only partial protection against thermal and pH‐induced degradation, and the stabilization was more pronounced for copper‐chlorophyllin than for native pigment, confirming that smaller or metal‐stabilized derivatives interact more effectively with CDs (Lombardelli, Benucci, Pippolini, Marabottini, and Esti 2025). Mechanistic studies further support this interpretation, showing that CDs can shield chlorophylls from oxygen and light to some extent but cannot prevent the fundamental chemical transformations responsible for color loss, especially Mg‐dechelation at acidic pH (Table 3).

Overall, CDs offer limited and matrix‐dependent stabilization for chlorophylls, with meaningful benefits only for certain derivatives (e.g., copper‐chlorophyllin), while native chlorophylls remain poorly protected due to their size, hydrophobicity, and intrinsic chemical lability (Kelanne et al. 2024). Compared to previous studies that primarily report stabilization outcomes, this analysis highlights the structural limitations governing CD–chlorophyll interactions, showing that steric mismatch and Mg‐center instability fundamentally constrain the effectiveness of inclusion complexation. This provides a mechanistic explanation for the consistently lower performance of CDs for chlorophyll stabilization, compared to other pigment classes.

#### 3.1.3 Flavonoids

Flavonoids are secondary polyphenolic metabolites widely distributed in the plant kingdom. They are present in leaves, flowers, fruits, and seeds and in plant‐derived foods such as fruits, vegetables, tea, wine, and cocoa (Tariq et al. 2023).

Physiologically, flavonoids contribute to flower pigmentation and aroma, promoting pollinator attraction, and play key roles in plant defense against abiotic and biotic stress. They act as UV filters, phytoalexins, and signaling molecules (B. Li et al. 2021; Shomali et al. 2022; Ramaroson et al. 2022). Numerous studies also describe their health‐promoting effects in humans, supporting their classification as functional ingredients (Bellavite 2023).

Structurally, flavonoids share a diphenylpropane (C6–C3–C6) skeleton, composed of two aromatic rings (A and B) linked by a three‐carbon bridge that may form a heterocyclic oxygen‐containing ring (C). Variations in ring C and in the attachment position of ring B determine their classification into: (i) isoflavones (Position 3), (ii) neoflavonoids (Position 4), and (iii) flavones, flavonols, flavanones, flavanonols, flavanols, anthocyanins, and chalcones (Position 2; Karak 2019).

Anthocyanins are water‐soluble pigments responsible for red, purple, and blue colors in many plants and fruits, mainly located in outer cell layers of berries and red grapes (Panche et al. 2016). Despite their recognized health benefits and use as natural colorants (Mozos et al. 2021; Gonçalves et al. 2021), they show limited chemical stability, strongly influenced by pH. Due to their ionic structure, they undergo pH‐dependent structural transformations with different color expressions (Roy and Rhim 2021).

Besides pH, temperature, light and oxygen significantly affect anthocyanin stability by promoting enzymatic oxidation and degradation (Aprodu et al. 2020; Kim et al. 2021; Sendri et al. 2023).

Anthocyanins can also undergo co‐pigmentation, forming complexes with colorless organic compounds or metal ions, which enhance color intensity and improve stability, sometimes more effectively than encapsulation (Tan et al. 2021). Indeed, the former typically involving polyphenols, flavonoids, organic acids, or other π‐conjugated molecules directly modifies the electronic environment of the anthocyanin chromophore through non‐covalent interactions, often producing pronounced hyperchromic effects (increased color intensity) and bathochromic shifts (changes toward longer wavelengths). In contrast, CDs primarily act by providing a protective microenvironment that limits the exposure of anthocyanins to water, oxygen, and other degrading agents. Therefore, while co‐pigmentation may offer superior color enhancement, CD encapsulation may provide complementary advantages related to solubility, physical stabilization, and delivery, suggesting that these approaches should be regarded as complementary rather than mutually exclusive stabilization strategies (J. Wang et al. 2024; Trouillas et al. 2016).

Anthocyanins are among the most chemically labile natural pigments, and their application in foods is limited by their strong pH‐dependent structural transformations, susceptibility to oxidation, and rapid degradation under light and heat. These weaknesses have motivated extensive research into CD inclusion complexes, which are particularly promising because the relatively small, aromatic structure of anthocyanins fits well into the β‐ and HP‐β‐CD cavities.

Computational molecular docking experiments and density functional theory calculations indicate that the host–guest complexation of anthocyanins is governed by an intricate thermodynamic balance dominated by hydrophobic effects, π–π stacking, and extensive hydrogen bonding networks (Su et al. 2024). Structurally, anthocyanins like cyanidin‐3‐glucoside and pelargonidin‐3‐glucoside comprise a flavylium cation core linked to one or more glycosidic moieties. Binding topology configurations show that the orientation of entry into the CD truncated cone depends directly on the substitution pattern of the guest. In complexes with native β‐CD, the planar, hydrophobic benzopyrilium core, and the attached B‐ring represent the primary guest segments, threading deeply into the apolar cavity (Ali et al. 2024). Concurrently, the bulky, highly hydrophilic glucoside branch is structurally restricted, anchoring at the outer secondary hydroxyl rim (Ali et al. 2024). When complexed with modified derivatives (e.g. HP‐β‐CD), the numerous peripheral hydroxyl groups of the anthocyanin form interlocking intermolecular hydrogen bonds with the functionalized host rim (Su et al. 2024). This specific spatial alignment and steric confinement inside the cavity play a dual role in stabilizing the guest pigment. First, the CD cone physically shields the electrophilic C2 position of the flavylium cation, preventing the nucleophilic attack of water molecules. This blocks the hydration reaction that would otherwise cause structural transformation into the colorless chalcone or carbinol pseudo‐base forms. Second, the localized confinement suppresses light‐induced degradation and limits thermal motion, raising the activation energy required for the disruption of the fully conjugated chromophore network (Pradhan et al. 2022).

Across real food matrices, however, the stabilizing effect of CDs is significant but not uniform. In beverages, for example, Aguilera et al. (2016) demonstrated that β‐CD complexation of black bean anthocyanins enhanced color intensity and slowed degradation during storage, especially in acidic sports drinks, although the improvement was strongly matrix‐dependent and diminished in low‐sugar formulations (Aguilera et al. 2016). Similar results were observed in model beverages enriched with purple sweet potato anthocyanins, where β‐CD increased thermal stability and reduced browning, yet co‐pigmentation alone sometimes produced stronger color enhancement, indicating that CDs protect primarily against oxidation rather than shifting chromatic equilibria (Quan et al. 2020). The best results were obtained when CDs were combined with whey or soy proteins, suggesting that CDs alone cannot fully prevent structural rearrangements and that multi‐component matrices offer synergistic stabilization (Quan et al. 2020).

Comparable evidence has been reported for chokeberry juice systems, where the addition of β‐CD significantly enhanced anthocyanin retention during thermal processing and long‐term storage. Howard et al. (2013) showed that β‐CD concentrations up to 3% markedly slowed pigment degradation and maintained color intensity in chokeberry juice stored for several months, demonstrating that CD complexation may enhance anthocyanin stability even under pasteurization and extended storage conditions. However, the magnitude of stabilization remained dependent on storage temperature and juice composition, confirming that matrix effects strongly influence the effectiveness of CD inclusion.

A recent study on strawberry anthocyanins complexed with β‑CD and starch (Ali et al. 2024) provides valuable mechanistic insight into the protective effects of CD‐based systems. The authors demonstrate that β‑CD improves resistance to heat, oxidation, and irradiation, compared to free anthocyanins, although starch‐based matrices outperform CDs under all stress conditions. This finding reinforces the notion that CD‐mediated stabilization is only one component of a broader protective architecture, and that physical entrapment and reduced oxygen diffusion can play an even more dominant role. Notably, the study is conducted in simplified aqueous systems rather than in real food matrices and therefore does not address key practical aspects such as sensory impact, matrix interactions, release kinetics, or regulatory feasibility. As such, while the work confirms the mechanistic relevance of CD inclusion, it also highlights the persistent gap between laboratory‐scale demonstrations and fully validated food applications.

More recently, the encapsulation of rose‐derived anthocyanins with β‐CD has been investigated for application in rose juice systems. The study of Xiong et al. (2025) demonstrated that CD complexation significantly improved thermal, oxidative, and photochemical stability, resulting in slower color degradation during storage, compared with the non‐encapsulated pigments. When incorporated into rose juice, the complexes maintained higher color intensity and extended pigment stability throughout storage, highlighting the potential of CD‐based encapsulation to enhance anthocyanin performance in real beverage matrices. At the same time, the authors observed that degradation still occurred over time, indicating that CDs mitigate but do not fully prevent anthocyanin breakdown in complex food systems.

Mechanistic studies further show that HP‐β‐CD forms stronger complexes than native β‐CD, leading to better protection against light and oxidation, although the degree of stabilization varies widely depending on anthocyanin acylation and glycosylation patterns (Mohammadalinejhad and Kurek 2021). Overall, the evidence indicates that CDs are highly effective in acidic beverages, but their stabilizing capacity is incomplete: anthocyanins still undergo pH‐driven structural changes, and long‐term stability under realistic storage conditions remains insufficiently studied. Major research gaps include systematic comparisons between CD encapsulation and co‐pigmentation strategies, evaluation of sensory impacts in real foods, and deeper investigation of how CD–anthocyanin complexes behave in complex matrices containing proteins, minerals, and competing polyphenols.

This interpretation goes beyond previous descriptive studies by linking stabilization efficiency to specific molecular features such as glycosylation and acylation patterns.

#### 3.1.4 Betalains

Betalains are water‐soluble nitrogen‐containing pigments derived from betalamic acid. From this core structure, two main classes originate: betacyanins, formed by condensation with cyclo‐L‐3,4‐dihydroxyphenylalanine and responsible for red‐purple colors; and betaxanthins, formed by conjugation with amino acids or amines, responsible for yellow‐orange hues. Their color depends on the extent of conjugation in the molecule, which determines the wavelength of absorbed light. Betacyanins absorb at longer wavelengths (532–550 nm) due to their larger electronic system, whereas betaxanthins absorb at shorter wavelengths (457–485 nm).

Because they generate a wide spectrum of colors, betalains are considered a promising alternative to synthetic dyes (Varelis et al. 2018; Calva‐Estrada et al. 2022).

Their stability in food applications is influenced by intrinsic and extrinsic factors including high temperatures, UV exposure, extreme pH (< 3 or > 7), oxygen, hydrogen peroxide (H_2_O_2_), metal ions, and degradative enzymes (Lombardelli et al. 2021; Sadowska‐Bartosz and Bartosz 2021).

From an application perspective, betalains are a technologically advantageous natural colorant, showing good stability between pH 3 and 7, which makes them suitable for low‐acidity foods such as dairy products, where anthocyanins are less stable (Djabali et al. 2024).

Beyond coloration, betalains have attracted attention for their health‐related properties, including inhibition of lipid oxidation, antioxidant and free radical‐scavenging activity, and anti‐inflammatory effects, suggesting a role in protection against oxidative stress–related disorders (Nirmal et al. 2023).

CDs have been explored as encapsulating agents due to the relatively small size and hydrophilicity of betalains, which allow more efficient cavity inclusion than in the case of bulky carotenoids or chlorophylls. Quantitative molecular docking analyses and semi‐empirical quantum mechanics indicate that the complexation thermodynamics of betalains are prominently driven by an optimization of host–guest steric fit, hydrophobic forces, and peripheral hydrogen bonding (Matencio et al. 2020). In inclusion complexes formed with native β‐CD, the aromatic, less polar aglycone region of betanin threads into the hydrophobic central cavity, while its bulky, highly hydrophilic glucoside branch extends outward, establishing an anchoring interface at the secondary hydroxyl rim (Cao et al. 2026). Conversely, docking simulations for custom betalain derivatives reveal that modified hosts like HP‐β‐CD or M‐β‐CD provide superior pocket geometry for stabilizing complex heterocyclic rings, such as indoline architectures, via localized van der Waals contacts and precise directional alignment (Matencio et al. 2020). This structural encapsulation provides excellent functional preservation by shielding the inherently fragile azomethine conjugated system of the betalain backbone. By encapsulating this core inside the truncated cone, the host restricts the accessibility of surrounding bulk water molecules, effectively reducing localized water activity and blocking water‐mediated nucleophilic attacks that induce hydrolytic cleavage into betalamic acid and cyclo‐DOPA (Cao et al. 2026). Furthermore, the tight steric confinement suppresses heat‐induced decarboxylation and dehydrogenation pathways, significantly delaying thermal discoloration during processing (Yang et al. 2024).

Tutunchi et al. (2019) applied β‑CD‑enhanced ultrasound extraction to red beet, reporting higher betanin and phenolic recovery and noting that the presence of β‑CD during extraction contributed not only to improved pigment yield but also to greater stability of the resulting extracts in beverage and gummy‑candy models. In their study, the authors attribute this dual benefit to the ability of β‑CD to interact with beet pigments during extraction, supporting both their recovery and their subsequent stability in food systems. This work therefore illustrates how β‑CD‑assisted ultrasound extraction can simultaneously enhance extraction efficiency and promote the stability of red beet compounds in practical food applications.

Complementary mechanistic insight has been provided by Matencio et al. (2020), who investigated the formation of supramolecular complexes between betalain derivatives and several CDs, including M‐β‐CD and HP‐β‐CD. The study demonstrated the formation of stable 1:1 host–guest complexes that significantly improved pigment resistance to photo‐oxidative degradation and enhanced solubility in aqueous systems. However, the work was performed on purified betalain derivatives rather than natural beet extracts and did not involve real food matrices. As a result, while the findings confirm that CDs can interact strongly with betalain chromophores (likely through inclusion of the aromatic moiety derived from betalamic acid) the practical implications for food stabilization remain uncertain. In particular, the study does not address how these complexes behave in complex food environments where pH fluctuations, metal ions, and enzymatic activity may accelerate pigment degradation.

Despite these promising results, the number of real‐food studies remains limited, and most experiments rely on short storage periods or simplified matrices that do not reflect industrial conditions. Critical gaps include the lack of comparative studies between CD encapsulation and more established techniques such as spray‐drying with maltodextrin, limited sensory evaluation of CD‐betalain complexes in foods, and insufficient understanding of how CDs interact with metal ions and endogenous enzymes that catalyze betalain degradation.

A recent study developed a dual‐channel starch‐based smart film incorporating a betanin–β‐CD inclusion complex and berberine for shrimp freshness monitoring (Yang et al. 2024). The betanin‐β‐CD complex exhibited markedly improved thermal stability and controlled release, compared with free betanin, and its incorporation into a corn‐amylose matrix enabled real‐time colorimetric and fluorescent responses to volatile amines during shrimp storage at different temperatures.

Although this work clearly demonstrates the capacity of β‐CD to enhance the thermal stability and controlled release of betanin, its application is oriented toward intelligent packaging rather than direct food fortification. Consequently, the stabilizing role of CDs in this context is linked primarily to maintaining pigment functionality as a freshness indicator rather than preserving color or nutritional value within the food itself. Nevertheless, the study highlights an emerging application domain for betalain–CD complexes, suggesting that CDs may play a valuable role not only in pigment stabilization but also in the development of smart packaging systems capable of monitoring food quality during storage. Compared with the existing reports that focus mainly on extraction yield or short‐term stability improvements, the present analysis distinguishes between CD‐assisted extraction and true stabilization within food matrices, clarifying that the observed benefits are often context‐dependent and not solely attributable to host–guest complex formation.

## 4 Mechanistic Basis and Synergistic Role of CDs in Stabilizing Natural Pigments

This section moves beyond descriptive reporting of individual studies and establishes a unifying mechanistic framework to interpret CD–pigment interactions. Rather than treating stabilization outcomes as empirical observations, the analysis identifies the physicochemical determinants that govern inclusion efficiency and protective effects across different pigment classes. Across all pigment classes examined, the effectiveness of CD encapsulation emerges as a structure‐dependent and matrix‐sensitive phenomenon. Its performance is governed primarily by three interrelated factors: (i) steric compatibility between pigment structure and CD cavity size; (ii) dominant degradation mechanism affecting the pigment (oxidative, thermal, or pH‐driven); and (iii) interactions with food matrix components. While CDs consistently improve dispersibility and short‐term oxidative stability, they show limited resistance to high‐temperature processing and cannot prevent intrinsic chemical transformations such as Mg‐dechelation in chlorophylls or flavylium hydration in anthocyanins. These recurring patterns indicate that CD encapsulation should not be regarded as a universal stabilization strategy but rather as a selective approach whose efficacy depends strongly on the physicochemical context.

The stabilizing action of CDs results from a combination of mechanisms rather than from a single protective effect. Structural complementarity between the hydrophobic CD cavity and specific molecular regions of natural pigments enables partial or complete host–guest inclusion. When a pigment molecule is located within or near the CD cavity, its exposure to reactive species such as oxygen, pro‐oxidant metals, nucleophiles, and water molecules can be reduced. This microenvironmental shielding slows key degradation pathways, particularly oxidative and photo‐induced reactions, thereby improving pigment stability in aqueous systems (Fuenmayor et al. 2021).

A second stabilizing contribution derives from the restriction of molecular mobility. Encapsulation can reduce the conformational freedom of labile chromophores or polyene chains, lowering the probability of thermally induced rearrangements and degradation reactions. In anthocyanins, for instance, CD complexation may partially stabilize the flavylium cation by reducing hydration reactions that convert it to the colorless hemiketal form, thereby contributing to improved color stability in acidic media (Quan et al. 2020). In this sense, the inclusion complex alters both the pigment microenvironment and the kinetics of degradation reactions, generating stabilization effects that exceed simple physical entrapment.

The synergistic nature of CD encapsulation becomes particularly evident when compared with other encapsulation strategies. Spray drying with carbohydrate carriers such as maltodextrin mainly provides physical protection, but it does not fully prevent oxygen diffusion into the matrix. In contrast, CD inclusion can reduce oxygen accessibility at the molecular level, slowing radical‐mediated degradation reactions (Yildiz et al. 2023). Co‐pigmentation, another common stabilization strategy for anthocyanins, enhances color intensity through intermolecular stacking interactions but offers limited protection against oxidative degradation. CD complexation, by contrast, improves pigment stability primarily by modifying the pigment microenvironment and reducing exposure to reactive species (Aguilera et al. 2016).

The extent of this stabilization is strongly pigment‐dependent. Small, relatively polar pigments such as anthocyanins and betalains fit well within β‐ or HP‐β‐CD cavities, allowing the formation of relatively stable host–guest complexes. Xanthophylls may also benefit from partial inclusion and hydrophobic shielding, whereas larger pigments such as chlorophylls and carotenes exhibit limited cavity compatibility and therefore weaker stabilization. This structure–function relationship helps explain why CDs outperform other encapsulation approaches for certain pigment classes but provide only moderate protection for others. In detail, for anthocyanins and betalains, available comparative studies suggest that CD‐based systems may outperform several conventional encapsulation approaches in terms of aqueous stability and color retention. However, for carotenoids and chlorophylls, direct head‐to‐head comparisons remain scarce, preventing definitive conclusions regarding the relative performance of native CDs.

Additional mechanistic insight can be obtained from studies employing crosslinked CD architectures such as CD nanosponges (CD‐NSs). These three‐dimensional networks contain multiple CD cavities interconnected within a polymeric structure, creating hydrophobic domains capable of retaining small molecules. Such systems may enhance pigment retention through a combination of cavity inclusion, hydrophobic interactions, and restricted molecular mobility (Salimpour and Malek 2021). However, these stabilization approaches cannot be attributed solely to classical host–guest inclusion, as non‐specific adsorption and network entrapment also contribute to the observed effects. This distinction highlights an important conceptual point: improvements observed in CD‐based systems may arise from multiple mechanisms depending on the architecture of the encapsulating matrix.

Recent studies have demonstrated the potential of CD‐NSs for improving the solubility, stability, and bioavailability of poorly water‐soluble bioactive compounds, particularly curcumin, resveratrol, oxyresveratrol and quercetin (Pyrak et al. 2024). Considering curcumin, enhanced performance has been attributed to the synergistic contribution of CD cavities and the interconnected porous architecture, which enables higher loading efficiencies and more sustained release profiles, compared with conventional inclusion complexes. These findings suggest that CD‐NSs may overcome some of the limitations associated with native CDs, especially when dealing with large or structurally complex molecules (Pyrak et al. 2024).

Evidence from real food matrices further supports the synergistic role of CDs. CD complexes often improve pigment dispersibility, reduce phase separation, and slow oxidative color loss, compared with non‐encapsulated pigments (Gomes et al. 2014). These effects reflect cooperative interactions between pigment molecules and the CD cavity that modify pigment reactivity and environmental sensitivity.

From a predictive perspective, optimal stabilization through CD encapsulation is expected when three conditions are satisfied: the pigment contains a molecular region compatible with the CD cavity diameter, degradation is dominated by oxidation rather than irreversible structural transformation, and processing temperatures remain below the threshold at which intrinsic chromophore breakdown occurs. Taken together, these observations demonstrate that CD‐based encapsulation operates through a mechanistically grounded synergistic framework in which structural complementarity, microenvironmental modulation, and multi‐pathway protection collectively enhance pigment stability beyond what is typically achieved through conventional encapsulation techniques.

Taken together, these observations confirm that CD encapsulation should not be interpreted as a universally effective strategy but as a mechanistically selective approach whose performance can be rationalized and, to some extent, predicted. This perspective directly addresses the gap identified in Section 1.6 by moving from descriptive reporting to a conceptually integrated interpretation of CD–pigment systems.

## 5 Conclusion

In this review, the main scientific studies on the use of CDs for the encapsulation of natural pigments have been collected and analyzed, with particular focus on their application as colorants in the food sector. Encapsulation techniques involving CDs have been shown to offer an effective solution to overcome the well‐known limitations of natural pigments, such as poor stability, low solubility, and limited bio‐accessibility after extraction from their original matrix.

Moving beyond conventional literature overviews, the present review proposes a mechanistic and comparative framework to interpret CD–pigment interactions, integrating structure–function relationships, dominant degradation pathways, and matrix effects. This approach allows the performance of CD encapsulation to be rationalized and, to some extent, predicted across different pigment classes. In particular, the stabilization achieved through CD encapsulation is interpreted as the result of a synergistic interplay of mechanisms (including cavity inclusion, microenvironmental shielding, and restriction of molecular mobility), which collectively contribute to reducing pigment degradation through multiple concurrent pathways rather than through a single protective effect.

The most commonly used technique involves the formation of inclusion complexes due to its ease of implementation, high protective efficacy, and good compatibility with a wide range of food matrices. In particular, the β form was found to be the most widely used among the CDs, owing to its favorable chemical structure and low cost. Notably, β‐CD accounts for approximately 70% of global CD production. Overall, the findings confirm the key role of β‐CD in the development of more water‐soluble, stable, and functional natural colorants, reinforcing the potential for its increasing and sustainable use not only in the food industry.

Despite the progress made, some aspects still remain to be clarified. In fact, much of the research is based on in vitro models, while in vivo studies are needed to verify the real bioavailability and benefits of encapsulated pigments. Furthermore, only a few works have evaluated the effect of inclusion complexes in real and complex foods. It would therefore be advisable to extend the studies to other types of products, in addition to simple drinks or yogurt, so as to gain a better understanding of their technological performance under industrial conditions. Another limitation concerns sensory evaluations: No study has explored how encapsulation affects flavor, aroma, texture, and, consequently, consumer acceptance, despite this representing a fundamental step for the adoption of pigments as natural dyes. Additional research is also needed to quantify cost–benefit ratios, evaluate scalability under industrial processing conditions, and systematically compare CDs with alternative encapsulation strategies. Moreover, a deeper understanding of interactions between CD–pigment complexes and food matrix components (proteins, lipids, and minerals) remains essential for real‐world applications. Filling these gaps is essential to fully exploit the potential of CDs, which have already demonstrated great application prospects. Overall, this review reframes CD encapsulation as a mechanistically selective and predictively interpretable strategy, providing a conceptual basis for the rational design of stabilized natural colorants in complex food systems.

## Author Contributions

**Chiara Pippolini**: writing – original draft, data curation, investigation. **Ilaria Benucci**: conceptualization, methodology, supervision, validation, writing – review and editing. **Claudio Lombardelli**: data curation, writing – original draft, investigation. **Marco Esti**: conceptualization, methodology, supervision, validation, writing – review and editing.

## Conflicts of Interest

The authors declare no conflicts of interest.
